# e-Consent in UK academic-led clinical trials: current practice, challenges and the need for more evidence

**DOI:** 10.1186/s13063-023-07656-8

**Published:** 2023-10-10

**Authors:** E. J. Mitchell, D. Appelbe, A. Bravery, L. Culliford, H. Evans, A. J. Farrin, K. Gillies, K. Hood, S. B. Love, M. R. Sydes, P. R. Williamson, N. Wakefield, as part of the e-Consent collaborative group

**Affiliations:** 1 Nottingham Clinical Trials Unit, School of Medicine, Applied Health Research Building, University Park, Nottingham, NG7 2RD UK; 2 Oxford Trauma and Emergency Care, Kadoorie Research Centre, Nuffield Department of Orthopaedic, Rheumatology and Musculoskeletal Sciences, University of Oxford, Oxford, UK; 3 Imperial Clinical Trials Unit, School of Public Health, Imperial College London, Stadium House, 68 Wood Lane, London, W12 7RH UK; 4 Bristol Trials Centre, University of Bristol, Bristol Medical School, 1-5 Whiteladies Road, Bristol, BS8 1NU UK; 5 Clinical Trials Research Unit, Leeds Institute of Clinical Trials Research, University of Leeds, Leeds, LS2 9NL UK; 6 Health Services Research Unit, Health Sciences Building, University of Aberdeen, Foresterhill, Aberdeen, AB25 2ZD UK; 7 Centre for Trial Research, College of Biomedical & Life Sciences, Cardiff University, Neuadd Meirionnydd, Heath Park, Cardiff, CF14 4YS UK; 8 MRC Clinical Trials Unit at UCL, Institute of Clinical Trials and Methodology, UCL, 90 High Holborn, London, WC1V 6LJ UK; 9 BHF Data Science Centre, Health Data Research UK, 215 Euston Road, London, NW1 2BE UK; 10 MRC-NIHR Trials Methodology Research Partnership, Department of Health Data Science, University of Liverpool, Liverpool, UK

**Keywords:** Clinical trial, e-Consent, Consent

## Abstract

**Background:**

During the COVID-19 pandemic, in-person healthcare visits were reduced. Consequently, trial teams needed to consider implementing remote methods for conducting clinical trials, including e-Consent. Although some clinical trials may have implemented e-Consent prior to the pandemic, anecdotes of uptake for this method increased within academic-led trials. When the increased use of this process emerged, representatives from several large academic clinical trial groups within the UK collaborated to discuss ways in which trialists can learn from one another when implementing e-Consent.

**Methods:**

A survey of UKCRC-registered Clinical Trials Units (CTUs) was undertaken in April–June 2021 to understand the implementation of and their views on the use of e-Consent and experiences from the perspectives of systems programmers and quality assurance staff on the use of e-Consent. CTUs not using e-Consent were asked to provide any reasons/barriers (including no suitable trials) and any plans for implementing it in the future. Two events for trialists and patient and public involvement (PPI) representatives were then held to disseminate findings, foster discussion, share experiences and aid in the identification of areas that the academic CTU community felt required more research.

**Results:**

Thirty-four (64%) of 53 CTUs responded to the survey, with good geographical representation across the UK. Twenty-one (62%) of the responding CTUs had implemented e-Consent in at least one of their trials, across different types of trials, including CTIMPs (Clinical Trial of Investigational Medicinal Product), ATIMPs (Advanced Therapy Medicinal Products) and non-CTIMPs. One hundred ninety-seven participants attended the two workshops for wide-ranging discussions.

**Conclusion:**

e-Consent is increasingly used in academic-led trials, yet uncertainties remain amongst trialists, patients and members of the public. Uncertainties include a lack of formal, practical guidance and a lack of evidence to demonstrate optimal or appropriate methods to use. We strongly encourage trialists to continue to share their own experiences of the implementation of e-Consent.

**Supplementary Information:**

The online version contains supplementary material available at 10.1186/s13063-023-07656-8.

## Background

The expectations of the informed consent process for participants in a clinical trial are well documented in ICH-Good Clinical Practice [1], The Declaration of Helsinki 1964 [2] and “The Medicines for Human Use (Clinical Trials) Regulations 2004 (as amended)” [3]. For Clinical Trials of Interventional Medicinal Products (CTIMPs), these expectations can be summarised as:

- Participants must be provided with information on the nature, significance, implications and risks of the trial and the right to withdraw from the trial at any time. A contact point for further information must also be supplied.
- Participants must be provided information by interview with the investigator or a member of the investigating team (where possible, in person).
- Participants must be provided with access to information about the study.
- Consent must be recorded in writing, dated and either signed or otherwise marked by the participant or authorised person if patient is unable to consent.

Typically, this process involves providing a Participant Information Sheet as a paper document that describes the study, what is involved in participating, why the study is being undertaken and the risks, advantages and disadvantages of participation [4]. As daily tasks have moved increasingly online, the progression to using digital media and methods in clinical trials is a natural one and the case has been made for better use of electronic methods in clinical trials [5–7].

In September 2018, the United Kingdom (UK) Medicines and Healthcare products Regulatory Agency (MHRA) and the UK Health Research Authority (HRA) published a joint statement on seeking consent by electronic methods, commonly referred to as “e-Consent” [8]. In this statement, the expectations of the Informed Consent process were outlined in general terms making it clear that provided the requirements of Good Clinical Practice (GCP) were met, and the rights of the participant are not breached, then it is acceptable to use e-Consent. The US Food and Drug Administration (FDA) also published guidance on the use of electronic informed consent in 2015, describing their expectations when e-Consent is used in a clinical study [9]. Several software vendors have also published, or otherwise provided, practical guidance on how e-Consent can be implemented when using their software [10–13].

A review of electronic consenting in remotely conducted studies by Skelton et al. published in 2020 identified 18 studies that used e-Consent [14]. However, only four of the included studies were clinical trials, none of which were conducted in the UK. The definition of e-Consent in the included studies was wide, ranging from a mixture of face-to-face and online methods to completely remote online solutions. A narrative synthesis was undertaken by their team, which produced five key themes and recommendations for researchers using e-Consent: (i) accessibility and user-friendliness of e-Consent, (ii) user engagement and comprehension, (iii) customisability to participant preferences and demographics, (iv) data security and (v) impact on research teams. It is important to note those recommendations are based on health research per se and, whilst some of the recommendations could also be applied to clinical trials, there could be additional considerations for trials.

During the COVID-19 pandemic, which started in March 2020, in-person healthcare appointments were reduced. Therefore, trialists involved in trials that continued to recruit participants needed to consider remote methods for conducting clinical trials, including e-Consent. Although some clinical trials may have implemented e-Consent methods prior to the pandemic, anecdotally, the uptake for this method appeared to have increased within academic trials during and post-pandemic (personal communication within CTUs).

When the increased use of e-Consent emerged, representatives from several large academic clinical trial groups within the UK formed the e-Consent collaborative group to discuss ways in which trialists can learn from one another when implementing e-Consent. The authors of this paper form the e-Consent collaborative group and represent the UK Trial Managers’ Network (UKTMN) (Mitchell, Wakefield), the UKCRC-registered Clinical Trials Unit Network (Bravery, Evans, Culliford) and the MRC-NIHR Trials Methodology Research Partnership overall (Williamson) and with specific representation from two sub-groups: the Trial Conduct working group (Gillies, Hood, Culliford, Love) and Health Informatics working group (Appelbe, Farrin, Sydes).

**Fig. 1:**
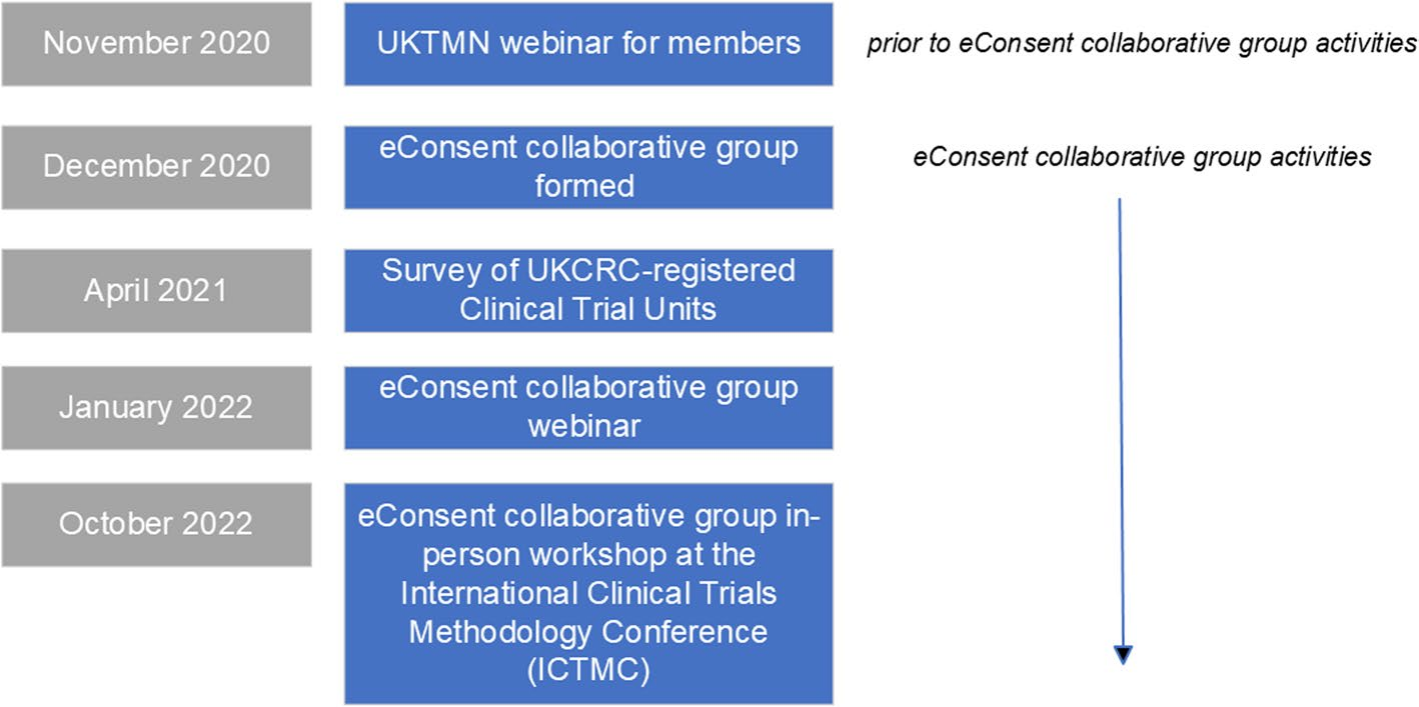
Timeline of the e-Consent collaborative group activities

This paper reports on several strands of work, including the results of a national survey of current practice in relation to e-Consent in UKCRC-registered Clinical Trials Units (CTUs) and key discussion points from two events held with the wider trials community (Fig. 1). Finally, we provide recommendations for practice and areas to be developed further via generation of evidence.

For clarity, we have focussed on the current UK regulatory definition of e-Consent: “The use of any electronic media (such as text, graphics, audio, video, podcasts or websites) to convey information related to the study and to seek and/or document informed consent via an electronic device such as a smartphone, tablet or computer” [8]. Importantly, our focus has been around the *recording* of consent using electronic methods, of people who *are joining* a trial as a participant. This paper does not focus on using electronic methods for the provision of information to *potential participants*.

## Methods

### Survey of UKCRC Clinical Trials Units on the use of e-Consent

To develop the questions to include in the survey, the literature and joint MHRA/HRA and FDA guidance on e-Consent were reviewed. Questions were agreed by members of the e-Consent collaborative group. The survey was initially designed on Microsoft Word®, for ease of review, and then built using the JISC online surveys^©^ tool (Supplementary Material 1). Questions were selected/developed to understand whether each responding CTU had already been using e-Consent and for what type of trials, and their views on the use of e-Consent and experiences from the perspectives of systems programmers and quality assurance staff on the use of e-Consent. CTUs not using e-Consent were asked to provide any reasons/barriers (including whether they had no suitable trials) and any plans for implementing it in the future. Free-text questions were included at the end of the survey to capture any other areas respondents felt were relevant to the topic. The usability and technical functionality of the questionnaire were tested by members of the group prior to the survey being made live. Adaptive questioning was used to elicit responses dependent upon a previous answer.

Our population is CTUs, based in the UK, who were fully registered within the UKCRC-registered CTU network, of which there were 53 in April 2021. An initial letter and link to the survey was disseminated via email to the CTU Directors in April 2021. The accompanying email explained that a single response was required from each CTU.

Respondents were asked to enter the name of the CTU whom they represented, to enable subsequent contact to be made with individual respondents, and to ensure only one response from each CTU was received. The name of the CTU does not otherwise contribute to the outputs from this survey. Three reminders were sent to CTUs, of which one was personalised to the CTU Director where a response had not been received from their CTU.

Data collection and analysis were led by the University of Oxford. Raw data were downloaded from the survey tool and stored as per University of Oxford policy, with only those involved in the analysis having access, and imported into Excel and NVIVO version 12 (QSR International, 2020) for analysis. The results are summarised using frequency counts and percentages.

### Workshops

In November 2020, prior to the formation of the e-Consent collaborative group, UKTMN held a webinar for its members (trial management staff and people working in trial conduct methodology). This was attended by 200 UKTMN members (maximum recommended for the online platform used) and presentations included (i) case studies from trial managers who had already implemented e-Consent and (ii) ethical considerations. Representatives from the HRA and MHRA attended and presented information included in the joint guidance.

It was clear from the initial UKTMN webinar that, whilst guidance is in place, there were many aspects of e-Consent that attendees were unclear about, particularly in relation to what was permissible from a regulatory perspective. Discussions following this webinar resulted in the e-Consent Collaborative Group being formed. It was agreed that whilst trial management staff play a significant role in the design and implementation of e-Consent, there are many other roles within trials that are also involved, including systems programmers, data management, and quality assurance staff. A subsequent online webinar was organised in January 2022 for the wider clinical trials community. Information about the webinar was disseminated by members within the collaborative group, using mailing distribution lists and social media. The aim of this event was to (i) provide information about the current regulatory requirements and guidance available, (ii) report on current practice across CTUs, (iii) provide practical examples when e-Consent has been implemented via two case studies (The VROOM Study [15] and the CO2 Study [16]), (iv) facilitate group discussion across different stages of the clinical trial lifecycle and (v) ascertain outstanding areas for methodological research into e-Consent. One hundred sixty-nine delegates attended the live webinar. To facilitate group discussions, online breakout rooms in MS Teams were used, with each breakout room representing a topic area, with ~ 20 participants in each. These topic area breakout rooms focussed on the following areas: (i) ethical considerations, (ii) participant acceptability, (iii) regulatory acceptability, (iv) implementation approaches, (v) programming/software, (vi) resourcing, (vii) quality assurance. Each breakout room was facilitated by 1–2 members from the e-Consent Collaborative Group. Key points from breakout discussions were then fed back to all webinar delegates during the next webinar session, with members of the e-Consent Collaborative Group responding to questions from delegates.

Finally, an in-person workshop was held at the 6th International Clinical Trials Methodology Conference (ICTMC) in October 2022. The purpose of this was similar to the previous online workshop: to share current practice and to enable delegates to discuss challenges and potential solutions to implementing e-Consent. The workshop was attended by 28 people across key roles including trial managers, quality assurance staff, programmers, data managers, methodologists and patient and public involvement representatives. The 3-hour workshop included presentations from trialists who have implemented e-Consent and discussion groups, who were split into the following topic groups: (i) technical aspects, (ii) participants and ethical aspects, (iii) regulatory aspects, (iv) site engagement and training. Groups were asked to discuss three questions about e-Consent in relation to their topic area: “What do we already know?”, “What don’t we know?” and “What are the outstanding questions?”. This was to enable mapping of the answers into areas that could be taken forward in the future, i.e. what can we put into guidance (*What we already know*), what we may need to seek clarification on (*What don’t we know*) and the evidence that needs to be generated (*What are the outstanding questions*).

Feedback and discussion points from the online and in-person workshops were entered live into a Padlet board (padlet.com, Wallwisher Inc.), and a subsequent visual mapping exercise was undertaken. Themes were derived from the direct feedback and the number of items under each theme counted based on the number of times they were mentioned across all discussions.

## Results

### Survey of UKCRC Clinical Trials Units on the use of e-Consent

**Fig. 2:**
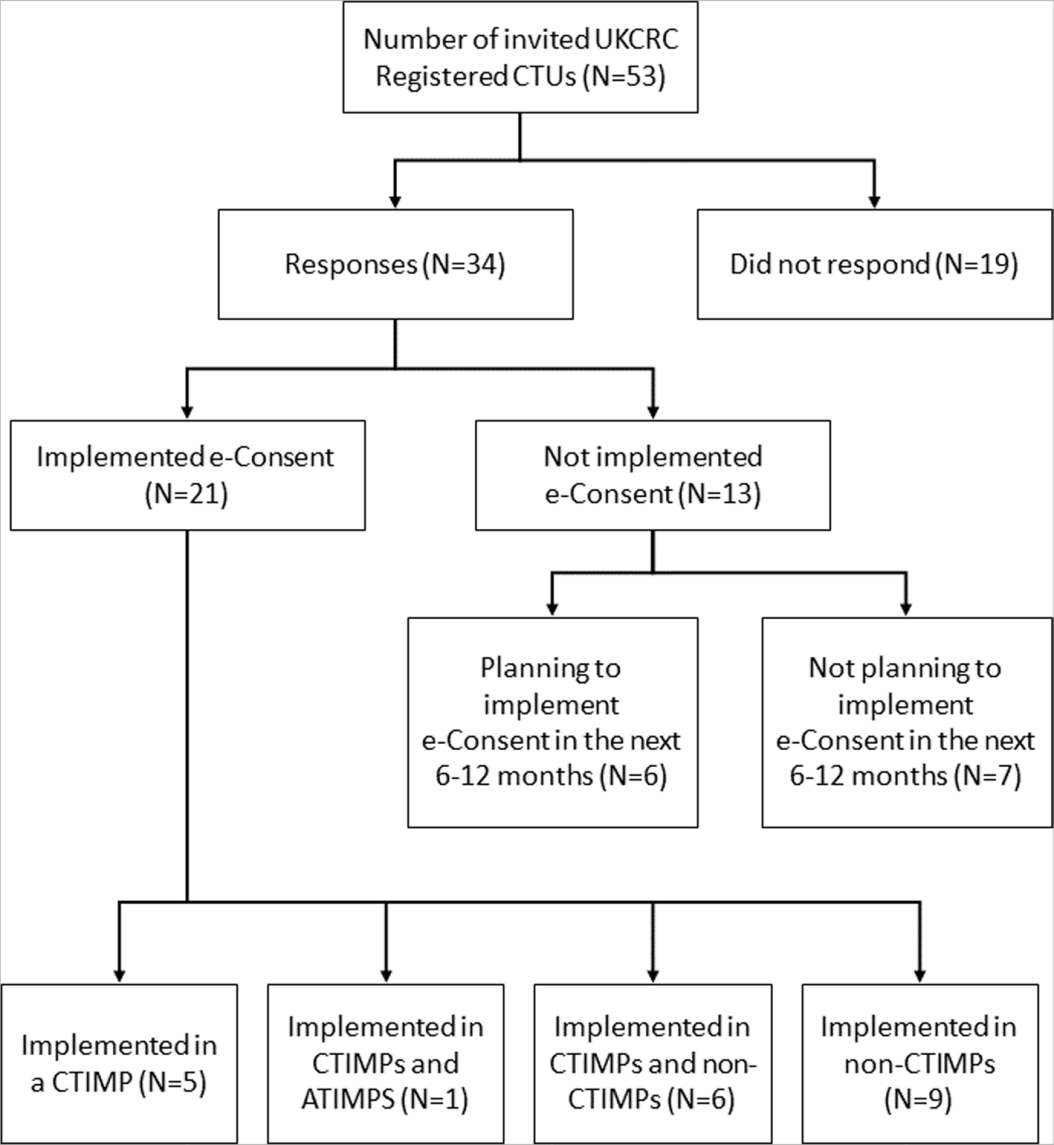
Summary of CTU responses to the survey

34/53 (64%) CTUs responded to the survey (Fig. 2), with good geographical representation across the UK. 21/34 (62%) CTUs had implemented e-Consent in at least one of their trials, across different types of trials, including CTIMPs (Clinical Trial of Investigational Medicinal Product), ATIMPs (Advanced Therapy Medicinal Products) and non-CTIMPs. CTUs were asked to record details for each of these types of trials, up to a maximum of four trials, providing details of 30 trials, including both adult and paediatric populations (Fig. 2). 13/34 (38%) of CTUs reported they had not implemented e-Consent. 6/13 (46%) of these CTUs, who did not plan to use e-Consent in the next 6 to 12 months, reported the following reasons: worried about regulatory issues (*N* = 1), would like more guidance before implementing (*N* = 3), worried about security (*N* = 1), lack of resource (*N* = 2), cost (*N* = 3), needing to know a tried and tested method is available (*N* = 2) and no suitable trials within the next 6–12 months (*N* = 1). More than one response could be selected.

The CTUs who had already implemented e-Consent in at least one trial were asked what resources they had used prior to implementing their e-Consent model. Responses included regulatory authority guidance (such as the MHRA/HRA joint statement on e-Consent [8]) and discussions with the clinical trial’s sponsor and staff at other CTUs.

**Fig. 3:**
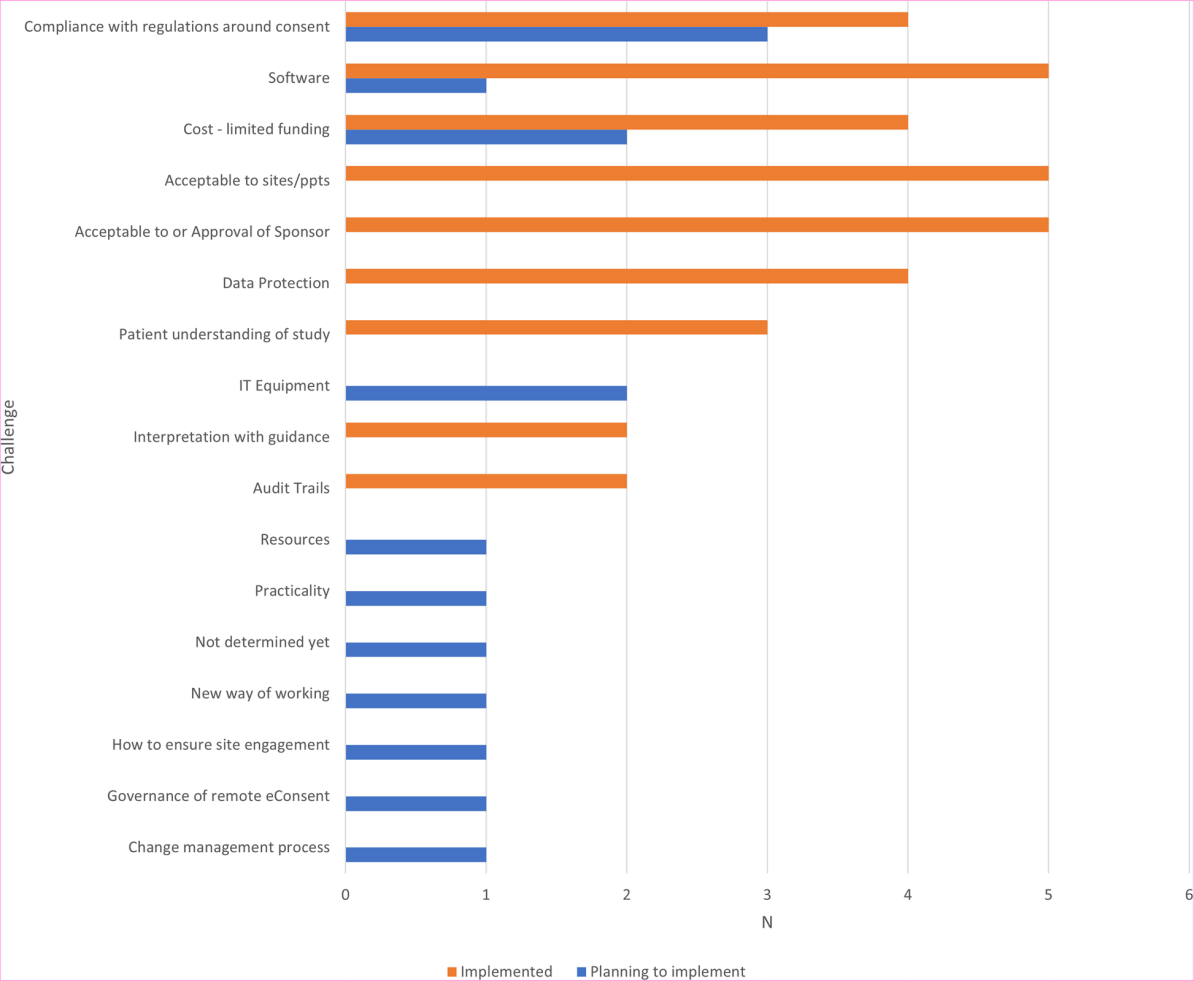
Key challenges foreseen when implementing e-Consent

CTUs were asked to report on the challenges they had foreseen or foresaw when implementing e-Consent (Fig. 3), with the most frequently reported challenge being compliance with clinical trial regulations.

The most used system for e-Consent was REDCap (Vanderbilt University) (Table 1), though CTUs reported using a range of systems. The validation of the different e-Consent systems was informed by a variety of guidance, ranging from CTU SOPs, through the MHRA/HRA joint statement [8] and other regulations such as eIDAS Regulation (EU) No 910/2014 (Digital signatures), resulting in either the use of validation plans or increased monitoring. CTUs reported that consent form data (including personal identifiable data) is usually handled according to the security models implemented on the server hosting the application and those programmed into the e-Consent software/server. Only five CTUs reported that consent data was encrypted at the time it was stored within the study database.

**Table 1:** Summary of systems used to record e-Consent

System	*N*	Ref/URL
REDCap	15	[10, 17]
Bespoke	6	N/A -
Qualtrics	2	[13]
Docusign	3	https://www.docusign.co.uk/
Meddidata RAVE	1	https://www.medidata.com/en/clinical-trial-products/clinical-data-management/edc-systems/
MedSciNet	1	https://medscinet.com/studies.aspx
OpenClinica (Participate Module)	1	https://docs.openclinica.com/3-1/participate/
REDCap Cloud	1	https://www.redcapcloud.com/
Sealed Envelope	1	https://www.sealedenvelope.com/

*NB*: with the exception of Docusign, all systems can be used to collect data

CTUs were asked to report if any questions had been posed during governance or regulatory reviews (e.g. Sponsor, Research Ethics Committee (REC) or regulatory review) relating to the proposed method of e-Consent. Most respondents (*N* = 15) had not received such questions. Where questions had been posed, they focussed on the following areas:

- Technical: Validation concerns [Sponsor].
- Conservatism: Concerns about being the first of their trials to implement [Sponsor].
- Technical: Information Governance/Data protection [Sponsor].
- Bureaucratic: Increased documentation/monitoring [Sponsor].
- Readability: Formatting [REC].

CTUs experienced in using e-Consent were asked if their e-Consent system(s) had been inspected by the MHRA; none had at the time of completion.

At the study level, CTUs were asked if, when developing the e-Consent process, they involved views of Patient and Public Involvement (PPI) representatives and/or trial sites. Responses were similar across all study types, with 17 trials involving their PPI representatives and 12 not, whilst six trials reported that they had not involved sites and 10 had (some trials were conducted remotely, without research sites). Whilst data on how acceptable participants found e-Consent was not collected in this study, CTUs reported that, anecdotally, sites liked the approach.

In the reported studies, several methods for the consent discussion process were used, including phone/video call and face-to-face with “e-” replacing paper (direct contact), and both paper and electronic options were available in some cases. Several methods had been used to verify the identity of the potential participant, including direct contact (in clinic (*N* = 17), phone/video call (*N* = 20)) and sending the participant an electronic link to their phone/email (*N* = 21) (CTUs could select more than one option).

For each nominated trial, CTUs were asked whether they had to provide additional equipment to sites to facilitate the use of e-Consent. Twenty-one studies (70%) responded that they had not, whilst seven (23%) had provided (or sites had purchased) additional equipment (e.g. tablet devices).

Ten responses were offered to a free-text question about areas CTUs felt required more research/guidance, not already covered by our survey. The most common was a need for more “detailed guidance”, “practical examples and shared experiences” and “expectations from regulators”.

### Webinar and workshop

**Fig. 4:**
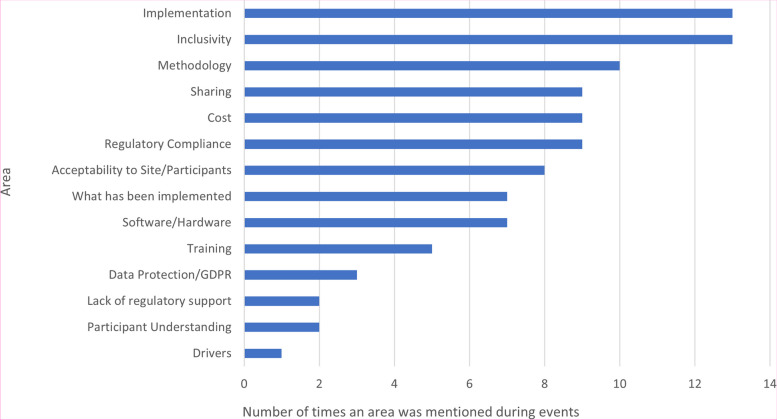
Key discussion points from online webinar and in-person workshops

Figure 4 summarises together the key discussion points from the online webinar (169 attendees) and the in-person workshop (28 attendees). Common themes arising included data security and clinical trial regulatory compliance, the acceptability of using e-Consent from a participant and site perspective, the choice of software and the costs associated with implementing e-Consent.

## Discussion

Our national survey has demonstrated that CTUs in the UK have been starting to implement e-Consent into clinical trials, including CTIMPs, within their portfolio, mostly to record an e-signature on a consent form or to send a consent form directly to a participant. It is also clear there are several reasons why CTUs have chosen to not implement e-Consent, at least in the short-term and in most trials. These were mainly due to the wish to receive more guidance prior to implementation and the cost, apparent cost or estimated cost of e-Consent solutions being prohibitive. The additional cost for the e-Consent software (whether as a stand-alone system or an add-on module to existing systems) varies, with the lower limit being £0 and the upper limit unknown (as this is commercially sensitive and depends on institutional arrangements with suppliers). The actual additional staff cost is unknown due to the degree of extra programming and validation that needs to be undertaken; obtaining this data will be difficult.

The challenges in implementing e-Consent identified in the survey (Fig. 3) and workshops (Fig. 4) were wide-ranging and consistent with discussions in subsequent events and themes included in the previous systematic review [14] and other recent surveys [18–22], particularly in relation to data protection/security and acceptability to sites and participants. Of note was ensuring regulatory/governance compliance, particularly in CTIMPs. This was not discussed within the 2020 systematic review; though as previously acknowledged, this reported on e-Consent in health research per se, rather than specifically in clinical trials, which are conducted in a far tighter regulatory environment. At the time of writing, the results of any MHRA inspections on trials implementing e-Consent have not been shared across the UKCRC-registered CTU network, so the implementation and acceptability of e-Consent systems in use to UK regulatory authorities remains unclear. It is anticipated that the findings of recent inspections can be shared by the appropriate study teams to aid in the improvement in the delivery of e-Consent across the CTU network.

The acceptability of patients must be a major consideration, as for any form of consent. Whilst the general response from patient representatives at the events reported here was positive, concerns around inclusivity (specifically relating to Digital Poverty [23], IT literacy and terminology and accessibility) were discussed at length. This is arguably one of the key messages from our work, in that it is important for trialists to consider the patient population and their access to electronic devices. During the COVID-19 pandemic, the use of electronic systems became more widespread; however, the digital divide [23] has since become more obvious. The use of e-Consent to record the discussion that has taken place (when based in a hospital/clinic) may well work, but if the participant does not have easy access to email, then sites will need to print out the consent form and ensure that the information sheets are also provided in paper format. For those that do have access to appropriate devices, the potential benefits for easily providing online material in different languages based on the end user’s client settings are wide-ranging, as are the benefits of ensuring that online media complies with “the Public Sector Bodies (Websites and Mobile Applications) (No. 2) Accessibility Regulations 2018” [24].

The acceptability to sites is easier to address, yet with the variety of different systems employed by UK CTUs, as demonstrated by our survey data, training and familiarity are areas that need to be addressed by CTUs and discussions with the site research teams at an early stage are recommended.

Numerous methodological questions were raised in the survey and events reported here, ranging from “do we just replicate the paper process electronically”, through to what works best for (potential) participants and should we be making use of the technology to aid the consent discussion further and determine the level of understanding by participants [25]. We encourage the trials methodology community to lead research into this area, generating evidence for future best practice.

At the operational level, there is a tremendous thirst to understand how other trials and CTUs have implemented e-Consent, and what lessons were learned, specifically in the cost of implementation (both upfront and ongoing cost and time), how the e-Consent solution (software) was arrived at, validation and training best practices and regulatory approval. The work reported here has helped to facilitate the sharing of information (in discussions, networking and the presentation of case studies) as has the work undertaken by Norwich CTU on REDCap [25]. A planned initiative underway by the CTU Network to provide operational implementation guidance will also help. Further literature reporting the successful implementation of e-Consent, drawing on a range of disciplines (including implementation science), would be welcomed.

Another operational concern raised was that of data security and compliance with the UK Data Protection Act and General Data Protection Regulations (GDPR). Specifically, this is related to storage and sharing of consent data, as frequently in e-Consent the consent form is made available to the participant digitally, rather than in paper form as is the case in non-e-Consent models. In the survey, most respondents stated that they had not encrypted the consent files when sending the completed consent form to the participant. This raises a risk due to the potential for data to be sent to the wrong person (the cause of ~ 15% of data security incidents reported to the UK Information Commissioners Office (ICO) up to Q2 2022 [26]), unless other steps have been taken to ensure that the contact details have been confirmed. There is also the risk that the consent forms or details of how to access electronic consent forms can be obtained if the participant’s mailbox is compromised. We recommend that detailed guidance on the operational best practice to secure the delivery of consent forms to participants be developed by the UK academic trials community.

Given the need for the global trials community to change practice due to the COVID-19 pandemic between 2020 and 2022 [27–29], the rate of change required to adapt to the changing clinical environment and the associated clinical trial recruitment pathways was paramount. It is encouraging that so many CTUs had already started to implement solutions to provide e-Consent. There are still many operational and methodological questions to be answered and best practice guidance to be developed, and without this evidence and guidance, there is reticence in the community to implement e-Consent in trials more widely.

We identify several strengths in this work and acknowledge limitations. In terms of strengths, we present the findings of a large collaborative effort, involving several leading national groups whose focus is on clinical trials and includes several strands of work. The national survey is the first report of current practice relating to e-Consent in clinical trials in the UK. In terms of limitations, the survey would have been optimised by a higher response rate, although this response rate is typical of surveys across UK CTUs [30–32]. We are unable to comment on the use of e-Consent in other jurisdictions on trials run by UK-based CTUs; however, guidance from other regulatory agencies [9] and researchers’ experiences [19, 21, 33, 34] suggest that the issues highlighted here are similar worldwide. The events that have been held to discuss the topic of e-Consent have included delegates from a range of clinical trial disciplines and patient and public involvement representatives from across the UK. Whilst we cannot report the exact number of individuals attending at least one event (including the initial UKTMN event, prior to the e-Consent collaborative group being formed), in total there were around 400 participants across all events.

We encourage trialists to share their own experiences of implementing e-Consent. Many trialists are still apprehensive about the implementation of e-Consent and require further guidance and support from teams where it has been implemented.

This is a starting point: there is still much to learn on this emerging topic. The next steps for our collaborative group are to focus on how trialists can best share information when they have implemented e-Consent, in order for others to learn, and build on, how to implement. To move forward, we have extended our collaboration to the European Forum for Good Clinical Practice (EFGCP) e-Consent Initiative (https://efgcp.eu/project?initiative=eConsent) where representatives from our group are involved with their academic workstream. Of note, the EFGCP e-Consent Initiative is focussing on the many digital components that could be included in the consenting process, including provision of trial information, rather than solely the *recording* of an electronic signature, which has been our focus to date. As previously shown in Fig. 4, there are many outstanding research questions relating to the acceptability and implementation and use of e-Consent. These will be discussed in future meetings of the MRC-NIHR Trials Methodology Research Partnership working groups. Finally, our group acknowledge there are gaps in both practical guidance and existing regulatory and ethical guidance and are taking a pro-active approach to providing feedback to the Medicines and Health Regulatory Agency (MHRA) and Health Research Authority (HRA) in the UK. Our collaborative group also plan on focussing on providing practical written guidance to the wider clinical trials community in the future. Whilst this guidance is taking shape, the links in Supplementary Material 2 may be of interest to the reader.

## Conclusion

e-Consent is increasingly being used, yet uncertainties remain amongst trialists, patients and members of the public. Many uncertainties are linked to a lack of formal, practical guidance and a lack of evidence to demonstrate the best and most appropriate methods to use. We strongly encourage clinical trialists to share their own experiences of implementation of e-Consent, either to a scientific journal or less formally by contacting a member of the collaborative group who may be able to facilitate sharing experiences via webinars, websites or blogs.

### Supplementary Information

**Additional file 1.** Survey questionnaire.

**Additional file 2.** Existing guidance.

## Data Availability

The dataset from the online survey is available from the corresponding author on reasonable request.

## Abbreviations

ATIMP: Advanced Therapy Medicinal Products
CTIMP: Clinical Trials of Interventional Medicinal Product
CTU: Clinical Trials Unit
EFGCP: European Forum for Good Clinical Practice
eIDAS: Electronic Identification and Trust Services
FDA: Food and Drug Administration
GCP: Good Clinical Practice
GDPR: General Data Protection Regulations
HRA: Health Research Authority
ICTMC: International Clinical Trials Methodology Conference
ICO: Information Commissioners’ Office
IT: Information Technology
JISC: Joint Information Systems Committee
MHRA: Medicines and Healthcare products Regulatory Agency
MRC: Medical Research Council
NIHR: National Institute for Health and Care Research
PPI: Patient and Public Involvement
REC: Research Ethics Committee
REDCap: Research Electronic Data Capture
SOP: Standard Operation Procedure
UK: United Kingdom
UKCRC: UK Clinical Research Collaboration
UKTMN: UK Trial Managers Network
