# Supplementary material for: e-Consent in UK academic-led clinical trials: current practice, challenges and the need for more evidence
Source: Trials. 2023 Oct 10;24:657. doi: 10.1186/s13063-023-07656-8 (PMC10565982; doi:10.1186/s13063-023-07656-8)
Supplement: Supplementary file 1 — Additional file 1. Survey questionnaire. [file 13063_2023_7656_MOESM1_ESM.docx]

**Joint TMRP Health Informatics Working Group, UKTMN & UKCRC CTU Network e-Consent Survey**

The use of eConsent in clinical trials is likely to be increasing, especially due to changes in recruitment pathways following the Covid-19 pandemic. We would like to understand:

- How many CTUs are currently using eConsent for clinical trials
- How many further CTUs are thinking about using eConsent for clinical trials
- For clinical trials where eConsent has been implemented, how it was implemented
- Where eConsent is still being considered, what are CTUs concerns and plans

The survey consists of several sets of questions that will need Trial Manager, Quality Assurance and Information Systems input, and are aimed at understanding both CTU level and trial level issues. The survey will take ~20 minutes for you to complete for trials units already using eConsent.

For the purpose of this survey, we use the MHRA definition of eConsent: “The use of any electronic media (such as text, graphics, audio, video, podcasts or websites) to convey information related to the study and to seek and/or document informed consent via an electronic device such as a smartphone, tablet or computer”.

If you use eConsent in multiple study types (ATIMP/CTIMP/non-CTIMP), please provide us with information on your eConsent implementation for each type of study.

Your responses will help us plan a joint meeting on this subject to showcase examples of solutions in practice and identify areas for further methodological research in this area.

| CTU Name |  |
| --- | --- |

| **CTU-level General Questions:** | | |
| --- | --- | --- |
|  | Does your CTU use any form of eConsent in any trials (or are currently integrating eConsent into an existing Clinical Trial)? | \|  \| Yes (Go to Q2) \| \| --- \| --- \| \|  \| No (Go to Q1.2) \| |
|  | Does your CTU plan to implement eConsent in any trials the next 6 to 12 months? | \|  \| Yes (Go to Q1.4) \| \| --- \| --- \| \|  \| No (Go to Q1.3) \| |
|  | Why not? | Choose all that apply   \|  \| Waiting for more mature technology \| \| --- \| --- \| \|  \| Worried about regulatory issues \| \|  \| Patient population may not wish to use eConsent approaches \| \|  \| Would like more guidance before implementing \| \|  \| Worried about security \| \|  \| Lack of resource \| \|  \| Cost \| \|  \| Need to know a tried and tested method is available \| \|  \| Other \| |
|  | If “Other” please specify |  |
|  | | |
|  | What preparatory work has your CTU done in determining how to implement eConsent? |  |
|  | What challenges does your CTU foresee the need to overcome? |  |

Survey flow:

- If CTU answers yes to 1.1 you progress below
- If CTU answers no to 1.2 survey ends after Q1.3, else end after Q1.5

| **CTU-level General Questions:** | | |
| --- | --- | --- |
|  | What preparatory work did your CTU undertake in determining how to implement your eConsent model? |  |
|  | What challenges did your CTU foresee the need to overcome? |  |
|  | Have you used eConsent in any Clinical Trials involving | Choose all that apply   \|  \| Paediatrics \| \| --- \| --- \| \|  \| Adults \| \|  \| Adults lacking Capacity \| |
|  | Do you use the same eConsent process across all of the current Clinical Trials in your portfolio? | Select one of   \|  \| Yes - All \| \| --- \| --- \| \|  \| Yes – Mostly \| \|  \| No \| \|  \| No – all Clinical Trials of the same type use the same model \| |
|  | Is there anything about eConsent that your CTU wished that you knew before implementation (or still wishes to know)? – If yes, please provide details |  |
|  | Is there anything your CTU would like to share with the wider UK community about your CTU’s implementation of eConsent? |  |

| **CTU-level Information Systems Questions:** These questions are aimed at understanding the technical solutions that have been implemented | | |
| --- | --- | --- |
|  | Please provide details of the eConsent systems in use at your CTU:   \|  \| System \| Feedback/Comments \| \| --- \| --- \| --- \| \|  \| Bespoke \|  \| \|  \| Castor \|  \| \|  \| ClinOne \|  \| \|  \| FireCREST \|  \| \|  \| Medidata RAVE \|  \| \|  \| REDCap \|  \| \|  \| REDCap Cloud \|  \| \|  \| Other \|  \| \|  \| Other \|  \| | |
|  | How does your CTU keep the consent form data secure (limit 250 words)? |  |
|  | How is the participant’s agreement to participate recorded (e.g. initials, checkboxes, typed name, signature) | Check all that apply   \|  \| Initials \| \| --- \| --- \| \|  \| Checkboxes \| \|  \| Typed Name \| \|  \| Signature \| \|  \| Other \|   If Other, please specify |
|  | If you collect signatures, how do you ensure that they are not used for any other purpose (e.g. not used in another form)? |  |
|  | Has your CTU experienced technological barriers to implementation? If so, please describe (or state ‘none’ if no barriers) |  |
|  | How did your CTU validate the process? |  |
|  | What guidance does your CTU use to ensure appropriate validation for the eConsent process? |  |
|  | Is there anything about the technical implementation and challenges of eConsent that your CTU wished (or still wish) that your CTU knew before implementation? |  |
|  | Is there anything your CTU would like to share with the wider UK community about your CTU’s implementation of eConsent? |  |

| **CTU-level Quality Assurance Questions:** | | |
| --- | --- | --- |
|  | Has your CTU’s eConsent process been inspected by MHRA (in general or for any specific trials) | Select one of   \|  \| Yes – unit and trial level \| \| --- \| --- \| \|  \| Yes – unit level \| \|  \| No \| \|  \| N/A – No trials inspectable (i.e. no CTIMPs) \| |
|  | If yes to the previous question, can you provide feedback on their comments? |  |
|  | What feedback did your CTU receive from regulatory bodies/Sponsor such as ethics/HRA (if applicable) when applying for approvals?   \|  \| Body \| Feedback/Comments \| \| --- \| --- \| --- \| \|  \| Ethics \|  \| \|  \| HRA \|  \| \|  \| Sponsor \|  \| \|  \| Other (UK) \|  \| \|  \| Other (non-UK) \|  \| | |
|  | How does your CTU document that the eConsent process you have implemented is fit for purpose? |  |
|  | What Guidance did your CTU use to check compliance with regulations? |  |
|  | How will eConsent records be archived? |  |
|  | Is there anything about the quality assurance of eConsent that your CTU wished (or still wish) that your CTU knew before implementation? |  |
|  | Is there anything your CTU would like to share with the wider UK community about your CTU’s implementation of eConsent? |  |

| **Trial-level Trial Management Questions**  *(Please complete this section for* ***an example*** *of* ***each type*** *of clinical trial your CTU has experiences with eConsent, thinking only about UK sites).*  This section will repeat, please answer all questions in this section for the same trial/study. | | |
| --- | --- | --- |
|  | What type of clinical trial are you reporting on in this example? | Select one of   \|  \| CTIMP \| \| --- \| --- \| \|  \| ATIMP \| \|  \| Other \| |
|  | Acronym/Short name for the study you are answering these questions for (if you are willing to share)? |  |
|  | What is the expected age range of the population that are involved in this clinical trial? | Choose all that apply   \|  \| < 18 \| \| --- \| --- \| \|  \| 18 – 60 \| \|  \| 60-70 \| \|  \| 70+ \| |
|  | What are the considerations that need to be taken into account around eConsent for the setting in which patients are to be recruited (e.g. WiFi access, Computer availability, tablet availability, computer literacy)? |  |
|  | What are you using eConsent to record trial? | Select all that apply   \|  \| Record of discussion \| \| --- \| --- \| \|  \| e-Signature \| \|  \| Sending Consent form to participant \| \|  \| Provision of PIS to participant \| \|  \| Paediatric Assent \| \|  \| Consent of patients lacking capacity \| |
|  | How is the consent discussion captured? | Select all that apply   \|  \| Face-to-face with “e” replacing paper \| \| --- \| --- \| \|  \| Via Phone/Video Call \| \|  \| No Researcher Contact \| \|  \| Other \| |
|  | If “Other” please specify |  |
|  | Is eConsent an additional / alternative option (to face-to-face) for obtaining consent in this study? | Select one of   \|  \| Yes – additional option \| \| --- \| --- \| \|  \| Yes – alternative option \| \|  \| No – only option \| |
|  | Did you involve your PPI partners in developing eConsent options for this study? | Select one of   \|  \| Yes \| \| --- \| --- \| \|  \| No \| |
|  | Did you involve staff at sites in the development of this process? | Select one of   \|  \| Yes \| \| --- \| --- \| \|  \| No \| \|  \| N/A – No Sites (Go to Q5.12) \| |
|  | In general, how have sites taken to your approach? Are there any notable exceptions? | (300 words max) |
|  | Did you discuss the e-Consent process with the trial sponsor? | Select one of   \|  \| Yes (Goto Q5.13) \| \| --- \| --- \| \|  \| No \| |
|  | What questions/comments/concerns did the sponsor have? |  |
|  | If you send the participant an electronic copy of the consent form, is it encrypted? | Select one of   \|  \| Yes (Goto Q5.15) \| \| --- \| --- \| \|  \| No (Go to 5.16) \| \|  \| N/A – Do not send (Go to 5.16) \| |
|  | If the consent form is encrypted, how does the participant receive the “key”? |  |
|  | How do you confirm the identity of the participant? | Select all that apply   \|  \| Face-to-Face in Clinic \| \| --- \| --- \| \|  \| Virtual Face-to-Face (e.g. video call) \| \|  \| Send an E-Mail with a link \| \|  \| Send an SMS with a link \| \|  \| Other \| |
|  | If “Other” Please Specify |  |
|  | How do you confirm that the person taking consent is on the delegation log? | Select all that apply   \|  \| Manual Check \| \| --- \| --- \| \|  \| Electronic check between systems \| \|  \| Ability to consent is enabled if they are on the delegation log when the researcher logs in to the system \| \|  \| N/A the participant is completing on their own \| \|  \| Other \| |
|  | If “Other” please specify |  |
|  | If Q5.10 = Yes/No  How do you ensure that the site stores a copy of the eConsent form (if applicable) and how do you monitor this? |  |
|  | Does the eConsent process mimic “paper” i.e. the eConsent form replicates a standard consent form and was sent to the participant in its entirety? | Select one of   \|  \| Yes (Goto Q5.22) \| \| --- \| --- \| \|  \| No \| |
|  | Please provide details of the approach you took? |  |
|  | Has eConsent helped or hindered recruitment into this trial? | Select one of   \|  \| Helped (Goto Q5.24) \| \| --- \| --- \| \|  \| Hindered (Goto Q5.24) \| \|  \| Not Sure \| |
|  | Please provide details |  |
|  | Do you have any information as to the effect of eConsent on participant follow-up? | Select one of   \|  \| Yes (Goto Q5.26) \| \| --- \| --- \| \|  \| No \| |
|  | Please provide details |  |
|  | If Q5.10 = Yes/No  Did you have to provide additional equipment (e.g mobile devices) to site to facilitate this process? |  |
|  | Is there anything about eConsent from an implementation and training perspective that you wished (or still wish) that you knew before implementation? | Select one of   \|  \| Yes (Goto Q5.29) \| \| --- \| --- \| \|  \| No \| |
|  | Please Specify |  |
|  | Is there anything you would like to share with the wider UK clinical trials community about the implementation of eConsent in the trial you manage at sites (e.g. how sites have responded/adapted to it’s use)? | Select one of   \|  \| Yes (Goto Q5.31) \| \| --- \| --- \| \|  \| No \| |
|  | Please Specify |  |

Add another Study/Trial Yes/No? – If yes, repeat section 5
