# Supplementary material for: e-Consent in UK academic-led clinical trials: current practice, challenges and the need for more evidence
Source: Trials. 2023 Oct 10;24:657. doi: 10.1186/s13063-023-07656-8 (PMC10565982; doi:10.1186/s13063-023-07656-8)
Supplement: Supplementary file 2 — Additional file 2. Existing guidance. [file 13063_2023_7656_MOESM2_ESM.docx]

| **Description** | **Source** |
| --- | --- |
| Implementing eConsent in REDCap | <https://norwichctu.uea.ac.uk/econsent/> |
| The TransCelerate eConsent Initiative aims to create general awareness and enable broad, voluntary implementation of eConsent. The eConsent Initiative has developed practical guidance and tools describing potential implementation considerations and eConsent components. | <https://www.transceleratebiopharmainc.com/assets/econsent-solutions/supporting-tools-resources/> |
| The aim of this document is to serve as an implementation guide when transitioning to electronic informed consent (eConsent) in clinical trials. The main purpose of the implementation guide is to provide a background of the benefits, processes and nuances of eConsent and offer practical advice to facilitate implementation | <https://www.eucrof.eu/> |
| MHRA/HRA Joint statement | <https://www.hra.nhs.uk/about-us/news-updates/hra-and-mhra-publish-joint-statement-seeking-and-documenting-consent-using-electronic-methods-econsent/> |
| Operational Considerations | Cobb NL, Edwards DF, Chin EM, Lah JJ, Goldstein FC, Manzanares CM, Suver CM. From paper to screen: regulatory and operational considerations for modernizing the informed consent process. J Clin Transl Sci. 2022 Mar 28;6(1):e71. doi: 10.1017/cts.2022.379. PMID: 35836789; PMCID: PMC9257776.  <https://www.ncbi.nlm.nih.gov/pmc/articles/PMC9257776/> |
